# Exploring the relationship between *OXTR* DNA methylation and temperament in children with neurodevelopmental disabilities

**DOI:** 10.3389/fpsyg.2026.1763938

**Published:** 2026-04-15

**Authors:** Eleonora Mascheroni, Niccolò Butti, Fabiana Mambretti, Laura Cordolcini, Annalisa Castagna, Vittoria Maucci, Andrea Citterio, Alessandra Bettinelli, Rosario Montirosso

**Affiliations:** 1 Scientific Institute, IRCCS E. Medea, 0-3 Center for the at-Risk Infant, Bosisio Parini, Lecco, Italy; 2 Scientific Institute, IRCCS E. Medea, Molecular Biology Lab, Bosisio Parini, Lecco, Italy; 3 Scientific Institute, IRCCS E. Medea, Specialist Functional Rehabilitation Unit, Bosisio Parini, Lecco, Italy

**Keywords:** DNA methylation, epigenetics, neurodevelopmental disabilities, oxytocin, Surgency/Extraversion, temperament

## Abstract

**Introduction:**

Oxytocin plays a crucial role in socio-emotional development. DNA methylation (DNAm) of the oxytocin receptor gene (*OXTR*) has been associated with socioemotional functioning and individual differences in temperament, yet its role in children with neurodevelopmental disabilities (NDs) remains underexplored.

**Methods:**

The present study examined *OXTR* DNAm across 13 CpG sites within intron 1 in a sample of 24 children with NDs and 24 typically developing (TD) children aged 3–36 months. DNAm was assessed from buccal epithelial cells collected via salivary swabs. To account for intercorrelations among CpG sites, principal component analysis (PCA) was applied, yielding two components (PC1: 7 CpGs; PC2: 6 CpGs). Temperament was assessed through parent-report measures of Surgency/Extraversion, Negative Emotionality, and Effortful Control. Analyses controlled for age and developmental quotient (DQ).

**Results:**

Results indicated higher *OXTR* DNAm in children with NDs compared to TD children, specifically in PC2. Higher DNAm in PC2 was associated with lower Surgency/Extraversion in children with NDs, but not in TD children. No significant associations emerged for Negative Emotionality or Effortful Control.

**Conclusion:**

These findings provide preliminary and exploratory evidence of an association between *OXTR* DNAm and temperamental positive emotionality in children with NDs. *OXTR* DNAm may represent a potential epigenetic correlate of early socio-emotional development in this population.

## Introduction

Neurodevelopmental disabilities (NDs) encompass a wide range of clinical conditions, including cerebral palsy and developmental delay. These disorders, whether congenital or acquired, may affect the brain or the neuromuscular system, leading to functional impairments that vary in severity and evolve differently over time (Morris et al., 2013; Weitzman et al., 2024). NDs may manifest independently or co-occur with other disorders, impacting various domains such as movement, cognition, hearing, vision, communication, and behavior (Olusanya et al., 2018; Tassé et al., 2020). Although children with NDs exhibit diverse etiological, genetic, and phenotypic characteristics, they commonly experience challenges in socio-emotional and behavioral regulation (Hauser-Cram and Woodman, 2016). For example, socio-emotional dysregulation has been observed in infants and children with developmental delay (Andrikos et al., 2024; Pears et al., 2015), cerebral palsy (Odding et al., 2006; Sigurdardottir et al., 2010), and autism spectrum disorder (White et al., 2014; Berkovits et al., 2017).

Both human and animal research highlight the essential role of oxytocin in shaping social traits and behaviors (Feldman et al., 2016; Hammock, 2015; Colonnello et al., 2017). Oxytocin is a neurohormone synthesized in the hypothalamus and released from the posterior pituitary gland that contributes toemotional regulation and social functioning (Fineberg and Ross, 2017). The brain’s oxytocin system is particularly sensitive to environmental influences during early development (Kompier et al., 2019). Genetic studies linked polymorphisms in the oxytocin receptor gene (*OXTR*) to socio-behavioral phenotypes and neural systems involved in social and affective processing (Aspé-Sánchez et al., 2016; Poore and Waldman, 2020; Tost et al., 2010; Yamasue et al., 2012). A recent literature review revealed that during childhood, *OXTR* variation is associated with socio-emotional and behavioral development and functioning, particularly in relation to autism spectrum disorder (ASD) (Kohlhoff et al., 2022). Specifically, *OXTR* polymorphisms have been linked to ASD symptom severity, social abilities, and neural mechanisms underlying social cognition. Including social communication, social responsiveness, and interpretation of social cues, and may also influence oxytocin system functioning by modulating social reward processing and emotional regulation (LoParo and Waldman, 2015; Inoue et al., 2010; Tost et al., 2010; Gong et al., 2017; Chander et al., 2022).

In addition to genetic variation epigenetic mechanisms have been increasingly investigated as key regulators of oxytocin system development (Ellis et al., 2021; Kraaijenvanger et al., 2019). Among these epigenetic mechanisms, DNA methylation (DNAm) involves the addition of a methyl group to cytosine within CpG sequences and caninflue gene expression and neural development (Shahrokh et al., 2010). In human epigenetic research, *OXTR* DNAm has been most commonly investigated within CpG-rich regions located in the promoter and intron 1 of the gene, which are involved in the regulation of *OXTR* transcription and gene expression (Kusui et al., 2001). Although DNAm is typically assessed in peripheral tissues in developmental studies, which may not fully reflect brain-specific epigenetic processes, accumulating evidence suggests a meaningful correspondence between peripheral and brain DNAm patterns (Braun et al., 2019; Gregory et al., 2009; Krol et al., 2019; Smith et al., 2015). Higher *OXTR* DNAm has been associated with associated with altered neural responses in regions implicated in emotional regulation, including the amygdala, fusiform gyrus, insula, temporal parietal junction, and anterior cingulate cortex (Dadds et al., 2014; Jack et al., 2012; Puglia et al., 2015). Importantly, from a developmental perspective *OXTR* gene expression in the human brain steadily rises during prenatal development and peaks in early childhood (Rokicki et al., 2022). Thus, this life period may represent a critical window for exploring oxytocin-mediated developmental processes, particularly in relation to socio-emotional development. Moreover, higher levels of *OXTR* DNAm are linked to socio-emotional outcomes also in clinical populations such as individuals with ASD (Gregory et al., 2009; Maud et al., 2018). However, to the best of our knowledge no study has investigated the link between OXTR DNAm and socio-emotional outcomes in children with NDs.

Temperament refers to biologically based individual differences that evolve over time through maturation and environmental influences, and plays a fundamental role in early social interactions (Kochanska et al., 2000). Rothbart’s model identifies three broad dimensions, Surgency/Extraversion, Negative Emotionality and Effortful Control, reflecting reactive and regulatory processes (Rothbart, 1981; Rothbart, 2011; Rothbart, 2012). Surgency/Extraversion reflects a high level of reactivity to positive stimuli, characterized by a tendency to seek excitement, engage in novel experiences, and show impulsivity and high activity levels; it is related to how easily a child becomes excited or active in response to external stimulation. Negative Emotionality is characterized by strong emotional reactivity responses such as fear, distress, and irritability and reflects how intensely a child reacts to discomfort or stress. Finally, Effortful Control refers to a child’s ability to regulate emotions and attention effectively. It encompasses attentional processes, such as the ability to shift focus, sustain attention, and orient to relevant stimuli, as well as behavioral regulation, which includes the capacity to inhibit impulses and manage responses appropriately (Rueda, 2012).

In early childhood, socio-emotional outcomes are more strongly associated with reactive dimensions such as Surgency/Extraversion and Negative Emotionality than with Effortful Control (Abulizi et al., 2017). Lower Surgency/Extraversion has been linked to reduced social engagement and resilience, whereas higher Negative Emotionality is associated with stronger emotional reactivity and difficulties recovering from distress (Laible et al., 2010; Dollar and Stifter, 2012; Perry et al., 2018). Importantly, the three core temperament dimensions proposed by Rothbart have been shown to be clinically relevant not only for normative socio-emotional development, but also for ND phenotypes. In children with NDs, lower levels of Surgency/Extraversion have been associated with reduced social engagement, diminished positive affect, and lower approach-related behaviors, particularly in ASD and in infants at high risk for ASD (Clifford et al., 2013; Korbut et al., 2022). Elevated Negative Emotionality, characterized by heightened emotional reactivity, irritability, and difficulties in recovering from distress, has also been consistently reported in children with developmental delay, ASD, and cerebral palsy, and is associated with greater emotion dysregulation and behavioral difficulties (Baker et al., 2002; Berkovits et al., 2017; Sigurdardottir et al., 2010). Finally, Effortful Control, reflecting emerging attentional and inhibitory capacities, appears to be compromised in several ND conditions, particularly in the presence of cognitive impairment or early neurological insult, and has been linked to difficulties in behavioral regulation and adaptive functioning (Konstantareas and Stewart, 2006; Rothbart and Bates, 2006).

Epigenetic variation has also been linked to temperament. For example, DNAm in the serotonin transporter gene (*SLC6A4*) has been associated with aspects of Surgency/Extraversion and Effortful Control in early infancy (Gartstein et al., 2016; Montirosso et al., 2016; Provenzi et al., 2021). DNAm of the glucocorticoid receptor gene (*NR3C1*) has been positively correlated with some dimensions of Negative Emotionality at 5 months (Ostlund et al., 2016). Additionally, during infancy, DNAm of brain-derived neurotrophic factor (*BDNF*) gene was associated with a progressive increase of Negative Emotionality between 3 and 6 months of age (Nazzari et al., 2024). As regards *OXTR*, it was found that increased DNAm was associated with higher Negative Emotionality during infancy (Nazzari et al., 2022; Krol et al., 2019). Recent epigenome-wide evidence also indicate that Surgency/Extraversion shows robust methylomic signatures, whereas associations with Negative Emotionality are weaker (Leri et al., 2024). Overall, the findings suggest that temperament-related epigenetic patterns emerge early in development, with Surgency/Extraversion demonstrating the strongest epigenetic associations.

### The current study

The current work aimed to investigate *OXTR* DNAm status in children with NDs and with typical development (TD) and its potential associations with temperament traits. The main purpose was to compare *OXTR* DNAm levels between children with NDs and with TD aged 3 to 36 months. Given that increased *OXTR* DNAm has been associated with developmental impairments (Gregory et al., 2009; Maud et al., 2018), we expected that children with NDs exhibit higher levels of *OXTR* DNAm relative to TD counterpart. Second, we examined whether *OXTR* DNAm influenced Surgency/Extraversion, Negative Emotionality and Effortful Control temperamental traits and whether these effects differed between children with NDs and TD. Given the evidence that epigenetic regulation of *OXTR* plays a key role in emotional regulation difficulties, which are partly rooted in early temperament traits (Rothbart, 2011; Rothbart, 2012), we hypothesized that higher *OXTR* DNAm would be associated with lower Surgency/Extraversion and higher Negative Emotionality in both groups. Given the broader role of the oxytocin system in socio-emotional regulation, we also explored whether OXTR DNAm was associated with Effortful Control (Yamasue et al., 2012). Additionally, we expected these associations to be more pronounced in children with NDs, given their heightened socio-emotional and behavioral challenges (Giusti et al., 2018). Considering the clinical heterogeneity in neurodevelopmental profiles among children with NDs, we included developmental quotient (DQ) as a control variable. As a proxy for general developmental functioning, DQ allowed for partial adjustment for broader cognitive and developmental differences that may be associated with both behavioral and biological outcomes.

## Materials and methods

### Participants and procedures

A total of 48 children, aged between 3 and 36 months, participated in this study. The clinical group included 24 children with ND who were recruited from the Neuropsychiatry and Specialist Functional Rehabilitation units of the Scientific Institute IRCCS “*E. Medea*” (Bosisio Parini, Lecco, Italy), where they were hospitalized alongside their mothers for assessment and rehabilitation. Inclusion criteria for the ND group required evidence of developmental delay, as documented by developmental scales (e.g., Griffiths Mental Developmental Scales Third Edition, Griffiths-III) and/or neurological examination indicating brain injury outcomes. Neurological evidence of brain injury outcomes referred to clinical findings documented in medical records, including structural brain abnormalities identified through neuroimaging (MRI), functional alterations detected by EEG, and neurological signs consistent with early brain injury evaluated during pediatric neurological examination. Developmental functioning was assessed using the Griffiths-III scales, which provide a standardized Developmental Quotient (DQ; mean = 100, SD = 15). According to clinical guidelines for the identification of developmental delay, scores between one and two standard deviations below the mean (DQ = 70–84) may reflect borderline or mild delay, whereas scores falling at least two standard deviations below the normative mean (DQ < 70) are considered indicative of significant developmental delay.

The TD group consisted of 24 children who were recruited through a convenience sampling method, with referrals from neonatologists, midwives, pediatricians, and nursery school educators in Lombardy (including the provinces of Monza-Brianza, Como, Lecco, and Milan). Inclusion criteria for the TD group included birth ≥ 37 weeks of gestation and the absence of any documented peri- or postnatal complications.

During a scheduled session, mothers from both groups completed a well-validated questionnaire to assess their children’s temperament Specifically, the Infant Behavior Questionnaire-Revised very short form (IBQ-R; Gartstein and Rothbart, 2003; Montirosso et al., 2011; Putnam et al., 2014) was administered for children aged 3–14 months, whereas the Early Childhood Behavior Questionnaire very short form (ECBQ; Putnam et al., 2006; Rothbart et al., 2009; Cozzi et al., 2013) was used for children aged 15–36 months (see Measures section below). Following questionnaire completion, children’s *OXTR* DNAm levels were collected through salivary swab collection by trained research staff according to the manufacturer’s guidelines. For both ND and TD groups, sample collection was performed after the completion of questionnaires and informed consent procedures, thereby ensuring comparable collection conditions across groups. Finally, children in the TD group were assessed using the Griffiths-III scales (Green et al., 2016; Stroud et al., 2016; Lanfranchi and Rea, 2017) to ensure consistency in evaluating developmental milestones across groups. For children with NDs, the Griffiths-III assessment was administered during the same hospitalization in which the research procedures were conducted, as part of the standardized clinical evaluation carried out by experienced clinicians specialized in neurodevelopmental assessment. Although the scores were retrieved from medical records, the assessment was temporally aligned with the research procedures.

This study received ethical approval from the Ethical Committee of the Scientific Institute IRCCS “*E. Medea*” (Prot. N. 09/2021) and was conducted in accordance with the Declaration of Helsinki. Written informed consent was obtained from all parents on behalf of their children. Saliva samples were collected exclusively to assess DNAm. Analyses were conducted at the Molecular Biology Unit of the Scientific Institute “*E. Medea*,” where samples are also stored. Immediately after collection, samples were pseudonymized using a coded identification system to ensure participant confidentiality; only the principal investigator and authorized research personnel had access to the key linking codes to participant identity. Samples are kept in restricted-access facilities for the duration of the project and subsequently archived in accordance with institutional biobanking procedures. Parents or legal guardians may withdraw consent for the use of biological samples at any time without any consequences.

### Measures

#### Sample characteristics

The clinical information of children with ND was collected from medical records. Clinical evaluations were conducted by a pediatric neurologist and/or a pediatric physiatrist and included medical examination and routine screening tests; the medical records also included the developmental assessment conducted using the Griffiths-III. For children with NDs, the Griffiths-III assessment was administered during the same hospitalization in which the research procedures were conducted, as part of the standardized clinical evaluation performed by experienced clinicians, and the resulting scores were subsequently retrieved from the medical records for the purposes of the present study. Within the clinical group, 12 children had a primary diagnosis of developmental delay (DD), while the remaining 12 were diagnosed with cerebral palsy (CP). Most children in both subgroups exhibited structural and/or functional brain abnormalities as evidenced by MRI and EEG findings (see Supplementary Table S1 for further clinical details on the two subgroups). For children with TD, developmental assessment was carried out by a trained research psychologist using the Griffiths-III scales during the scheduled study session. Sample characteristics are reported in Table 1.

**Table 1:** Sample characteristics.

	NDs (*n* = 24)		TD (*n* = 24)	
	*n*	*%*	*n*	*%*
Child sex				
Female	12	50	14	42
Male	12	50	10	58

	Mean	SD	Range	Mean	SD	Range
Child age (months)	18.96	8.73	3.4–36	16.37	11.46	3.4–36
Maternal age (years)	35.54	5.62	26–48	34.04	3.70	29–42
Child Developmental Quotient (score)	65.21	26.26	20–97	105.41	7.94	86–125

NDs, Neurodevelopmental disabilities; TD, Typical development.

The two groups were comparable in terms of child age and maternal age. As expected, children with NDs showed lower developmental quotient scores compared with TD children. The distribution of DQ scores across the two groups is illustrated in Supplementary Figure S1.

#### Infant temperament

For children aged 3 to 14 months, mothers completed the Italian version of the Infant Behavior Questionnaire-Revised very short form (IBQ-R very short form; Gartstein and Rothbart, 2003; Montirosso et al., 2011; Putnam et al., 2014). For children aged 15 to 36 months, temperament was evaluated using the Italian version of the Early Childhood Behavior Questionnaire very short form (ECBQ very short form; Putnam et al., 2006; Rothbart et al., 2009; Cozzi et al., 2013). The IBQ-R very short form and the ECBQ very short form consist of 37 and 36 item respectively, designed to evaluate temperament across three broad dimensions, that are continuous between the IBQ-R and ECBQ: Surgency/Extraversion, which is continuous with the factor of Positive Emotionality/Surgency; 2. Negative emotionality; 3. Effortful control, which is continuous with the factor of Orienting/Regulatory capacity. Each item is rated on a 7-point scale, ranging from 1 (“*never*”) to 7 (“*always*”), with an option to mark an item as not applicable if the described situation has never been observed. Scale scores are calculated by averaging responses across relevant items, yielding a possible range of 0–7, where higher scores reflect greater expression of the corresponding temperament trait. Although the two instruments include age-appropriate and partially distinct items and subscales, they are both grounded in Rothbart’s theoretical model (1981; 2011) and share the same overarching structure across the three temperament dimensions. Moreover, the use of the same rating scale and identical scoring procedures allows direct comparability of dimension scores across instruments, without the need for further transformation or standardization. This approach is supported by previous research showing consistency of results across IBQ-R and ECBQ in studies comparing temperament across early developmental stages and cultural contexts (e.g., Montirosso et al., 2011; Cozzi et al., 2013).

#### DNAm assessment

Infant buccal cells were obtained through salivary swabs for DNAm assessment using DNA Genotek Oragene OC-175, according to manufacturer guidelines. Biological samples were obtained by trained research assistants. DNA was extracted following manufacturer’s protocols and quantified on a Qubit 2.0 fluorometer (Invitrogen). Bisulfite conversion was performed on 200 ng of genomic DNA using the EZ DNA methylation lightning kit (ZymoResearch, Inc., Irvine, CA, United States). A portion of OXTR intron 1 (chr3: 8810654–8,810,919) was selected for analysis. Following prior research, we focused on this CpG-rich region because it has been repeatedly implicated in socio-emotional and neurodevelopmental phenotypes. In particular, Gregory et al. (2009) reported differential OXTR DNAm within intron 1 in individuals with ASD compared with controls, identifying several CpG sites located between −860 and −959 relative to the translational start site. Subsequent studies have examined overlapping CpG sites within the same intron 1 cluster and reported associations with social cognition, emotional processing, and stress-related outcomes (Puglia et al., 2015; Krol et al., 2019). This intron 1 CpG cluster is considered functionally relevant because it lies within a CpG island spanning exon 1 and intron 1, a genomic region involved in the regulation of OXTR transcription and gene expression (Kusui et al., 2001; Kumsta et al., 2013). Accordingly, this segment represents one of the most widely investigated candidate regions in the OXTR DNAm literature, particularly in studies addressing socio-emotional functioning and neurodevelopmental risk (Kraaijenvanger et al., 2019). Consistent with this literature, we assessed DNAm across 13 CpG sites within the OXTR intron 1 cluster. Several of these sites overlap with, or fall in close proximity to, CpG positions previously examined in studies of OXTR DNAm and socio-emotional phenotypes (Nazzari et al., 2022). To account for intercorrelations among neighboring CpG sites and reduce multiple testing, site-level DNAm variability was summarized using principal component analysis (PCA), an approach commonly adopted in candidate-gene DNAm research to capture shared methylation patterns across correlated CpGs (Cecil et al., 2014; Krol et al., 2019; Nazzari et al., 2022). Interpretation of CpG-level contributions was informed by prior literature on the relevance of specific intron 1 CpGs for socio-emotional and neurodevelopmental phenotypes (Gregory et al., 2009; Puglia et al., 2015). This target region was amplified, using primers designed with Bisulfite Primer Seeker (forward: TAGGTAGTTGGGTGTTAAGTAGGGGTGG; reverse: AGCCCAAACCCTAACATAAACACCTCC). Specific tails were added to the primers in order to allow synthesis of SureSelect-style libraries of target fragments. Libraries were purified with AMPure XP beads (Beckman Coulter) and quantified on a Bioanalyzer 2,100 (Agilent). A positive control was included at each step of the experiment, and the degree of methylation in this control sample was verified to be as expected. Furthermore, a negative control (a sample without DNA) was included in all experiments to monitor for potential contamination that could lead to false-positive results. To minimize technical biases, we used high quality input DNA and verified sample purity by measuring 260/280 and 260/230 absorbance ratios, we kept amplicon size short (<350 bp) to maintain sequence fidelity during PCR, we assessed library yield and fragment size distributions (using Bioanalyzer instrument) before sequencing. Aliquots of each indexed library were pooled and sequenced on a NEXTSeq 500 (Illumina Inc., San Diego, CA, United States) using a v2 Reagent kit, 300 cycles PE. Paired ends reads from each sample were independently aligned to all the reference sequences by a parallel striped Smith-Waterman algorithm. Only paired reads that aligned coherently to the same reference sequence were retained. At each CpG site in each sequence, the four base frequencies were evaluated and reported along with the C → T percentage. Methylation data, grouped in percentages and reads, were screened and pruned based on the number of reads for each subject, not taking into account all the values below 100 reads count. Positions of the selected *OXTR* CpG sites in human genome assembly GRCh37 (hg19), percentage and range for DNAm level in each CpG site in the two groups are shown in Table 2.

**Table 2:** Positions of the selected OXTR CpG sites in human genome assembly GRCh37 (hg19), percentage and range for DNAm level in each CpG site in the two samples.

CpG sites number	Position	NDs		TD	
		% DNAm	% DNAm range	% DNAm	% DNAm range
CpG1	chr3: 8810889–8,810,890	1.37	0.08–4.13	1.04	0.00–1.68
CpG2	chr3: 8810874–8,810,875	2.16	0.08–4.38	1.54	0.06–2.47
CpG3	chr3: 8810862–8,810,863	6.34	0.57–11.46	5.72	1.94–14.60
CpG4	chr3: 8810855–8,810,856	1.79	0.08–4.21	2.08	0.10–10.12
CpG5	chr3: 8810832–8,810,833	31.17	24.28–43.28	27.30	17.81–36.35
CpG6	chr3: 8810807–8,810,808	35.39	22.25–44.39	35.04	22.47–59.31
CpG7	chr3: 8810797–8,810,798	60.66	44.29–70.49	60.41	47.16–87.83
CpG8	chr3: 8810774–8,810,775	43.81	33.25–49.97	39.39	19.52–52.29
CpG9	chr3: 8810733–8,810,734	24.22	18.42–32.96	22.07	14.07–40.57
CpG10	chr3: 8810708–8,810,709	8.86	4.17–15.42	8.39	3.38–16.84
CpG11	chr3: 8810699–8,810,700	9.89	4.22–16.46	9.77	5.07–19.72
CpG12	chr3: 8810681–8,810,682	11.08	3.19–15.88	10.72	0.35–16.80
CpG13	chr3: 8810679–8,810,680	12.04	6.43–20.04	11.68	5.25–20.86

NDs, Neurodevelopmental disabilities; TD, Typical development.

**Figure 1:**
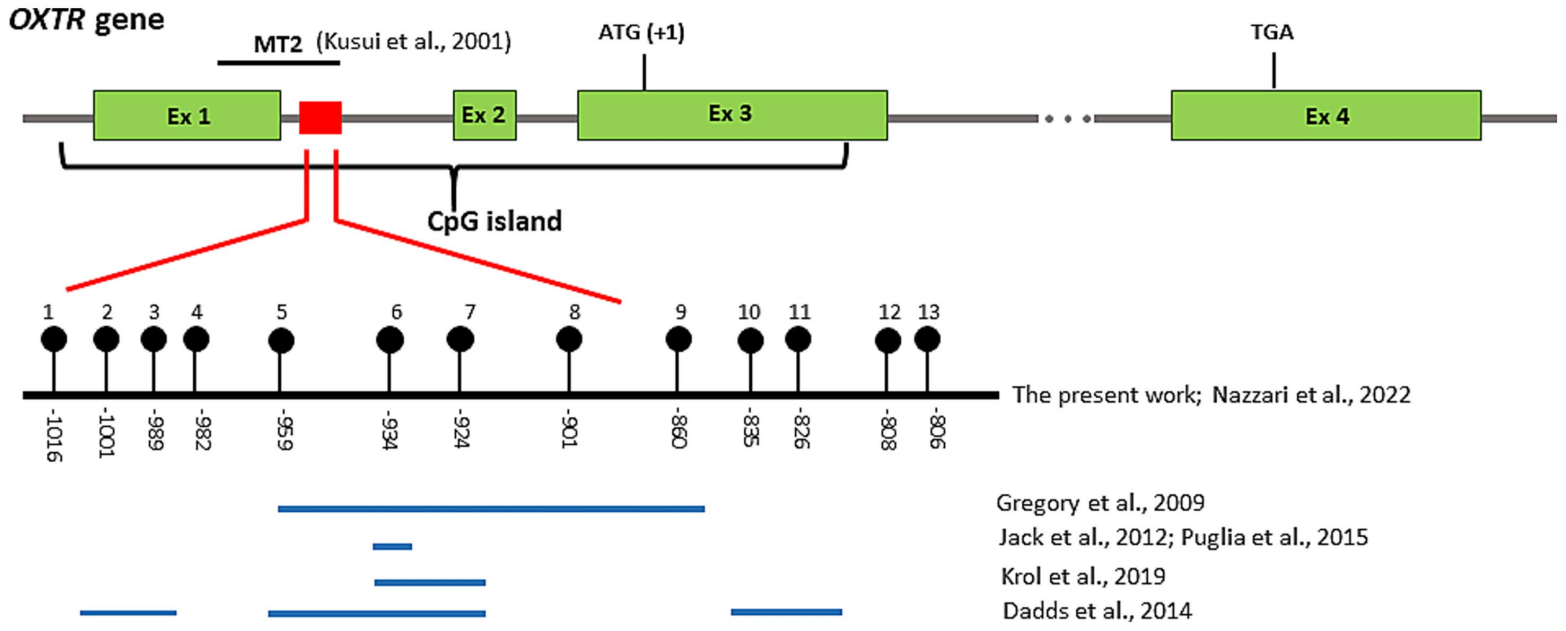
The CpG sites examined in the present study and in previous research. The OXTR gene spans 17 kb and is composed of 3 introns and 4 exons (green boxes). Exons 1 and 2 correspond to non-coding regions while exons 3 and 4 encode the amino acids of the oxytocin receptor (ATG denotes the translation start site and TGA the stop codon). The brace indicates the location of a CpG island, which spans exon 1 through exon 3. Kusui et al. identified a region within this CpG island (MT2) important for the regulation of tissue-specific gene expression. The red box in intron 1 indicates the location of the target region analyzed in the present study. This region is composed of 13 CpG sites (black circles), for which the CpG site number (top) and position relative to the translation start site (bottom) are shown. Some of these CpG sites have been analyzed in previous works (see text), as displayed by the blue segments.

The analyzed region has been previously linked to various socio-behavioral outcomes, including temperament traits (Kumsta et al., 2013; Nazzari et al., 2022). Figure 1 illustrates the location of the target region examined in the present study, as well as the CpG sites within intron 1 that have been analyzed in previous research.

### Analyses plan

#### Data reduction

To summarize DNAm variability across the 13 CpG sites within OXTR intron 1 and to reduce multiple testing, we applied principal component analysis (PCA) to CpG-level DNAm values. This approach is commonly used in candidate-gene DNAm studies because DNAm levels at neighboring CpG sites within the same regulatory region are typically correlated and may reflect shared regulatory influences rather than independent biological signals (Cecil et al., 2014; Smith et al., 2015). The PCA was conducted on the full sample (i.e., including both ND and TD children) to derive a common component structure across groups. Component retention was determined using parallel analysis (Horn, 1965), which compares observed eigenvalues with those obtained from randomly generated data and is considered a robust and conservative criterion for factor retention in epigenetic research (Hayton et al., 2004). Parallel analysis supported the retention of two components. A Varimax rotation was then applied to facilitate the interpretability of component loadings. Although the PCA was data-driven and not based on *a priori* hypotheses regarding specific CpG groupings, the resulting components reflect clusters of CpG sites located within intron 1, a region that has been repeatedly implicated in OXTR regulation and socio-emotional phenotypes (Gregory et al., 2009; Kumsta et al., 2013). Similar PCA- or composite-based approaches to summarizing DNAm across multiple OXTR CpG sites have been adopted in prior studies to capture shared variance and improve statistical power (Cecil et al., 2014; Krol et al., 2019).

#### Group comparisons

Preliminary analyses were conducted to examine potential group differences and relationships between sample characteristics (i.e., sex, age and DQ). First, chi-square tests were used to compare sex distribution between the NDs and TD groups and between the two clinical subgroups (cerebral palsy vs. developmental delay), while independent samples *t*-tests were performed to assess differences in age and DQ. Additional *t*-tests were conducted to examine potential sex differences in *OXTR* DNAm levels and temperament traits (Surgency/Extraversion, Negative Emotionality, and Effortful Control). To explore associations between age, *OXTR* DNAm, and temperament, Pearson’s correlation analyses were performed. Furthermore, preliminary correlations were computed to investigate the relationship between DQ and *OXTR* DNAm.

After these preliminary analyses and after verifying the normal distribution of the included variables, differences in *OXTR* DNAm levels in PC1 and PC2 as well as possible differences in temperament traits between children with ND and those with TD were analyzed using independent samples *t*-tests.

#### Moderation models

Moderation models were tested, including *OXTR* DNAm levels that significantly differed between NDs and TD as predictor, group (NDs vs. TD) as moderator, and child temperament traits as outcome variables. As previous research has identified differences between males and females in *OXTR* DNAm, the models were controlled for child’s sex (Nazzari et al., 2024). Additionally, since the sample included children with a broad age range (3 to 36 months) and differences in developmental level (20 to 125) and severity of developmental delay (20 to 97), the models were adjusted for age and Griffiths-III DQ. Controlling for DQ thus allowed us to account for individual variability in general developmental level, and to better isolate the specific contribution of DNAm and group to the observed effects on temperament outcomes. Because the IBQ-R and ECBQ are age-specific instruments designed to assess the same temperament dimensions across early development, and because instrument administration was fully determined by age, instrument type was not included as a separate covariate to avoid collinearity with age. Statistical analyses were carried using SPSS29 for Windows and PROCESS macro (Hayes, 2022) with alpha set at *p* < 0.05.

## Results

### Data reduction

The PCA (Horn, 1965) indicated that only the first two eigenvalues exceeded those obtained from randomly generated data suggesting the extraction of two factors (PC1: eigenvalue = 4.59 and PC2: eigenvalue = 2.70), setting a Varimax rotation, suppressing coefficients lower than 0.30 (Table 3). Sampling adequacy was acceptable (KMO = 0.767), and Bartlett’s test of sphericity was significant, *χ^2^* = 283.29, *p* < 0.001, supporting the suitability of the data for PCA. PC1 (composed of 7 CpG sites: CpG3, CpG4, CpG5, CpG6, CpG7, CpG10, and CpG11) and PC2 (composed of 6 CpG sites: CpG1, CpG2, CpG8, CpG9, CpG12, and CpG13) accounted, respectively, for 32.57 and 23.80% of the total variance in *OXTR* DNAm (cumulative variance explained = 56.37%) and were used for the analyses.

**Table 3:** Principal component analysis (PCA) on children’s OXTR DNAm levels conducted among the 13 CpG sites.

*OXTR* DNAm sites	Principal Components	
	PC1	PC2
CpG1		**0.532**
CpG2		**0.588**
CpG3	**0.546**	0.320
CpG4	**0.843**	
CpG5	**0.505**	0.498
CpG6	**0.821**	
CpG7	**0.842**	
CpG8		**0.793**
CpG9	0.358	**0.651**
CpG10	**0.794**	
CpG11	**0.821**	
CpG12		**0.689**
CpG13	0.324	**0.712**

Loadings on the respective principal components (PCs) are reported in bold. Loadings < 0.30 are not reported.

### Group comparisons

Preliminary analyses indicated no significant differences between children with NDs and TD children in terms of sex and age, whereas differences in DQ were observed. Within the ND group, no significant differences emerged between clinical subgroups (CP vs. DD) in *OXTR* DNAm or temperament. No significant associations were found between age and *OXTR* DNAm. Detailed results of preliminary analyses are reported in the Supplementary materials. A significant difference emerged between groups in PC2 [*t* (46) = 2.431, *p* = 0.019, Cohen’s *d* = 0.70]. Specifically, levels of DNAm in PC2 were significantly higher in children with NDs (*M* = 17.98%, SD = 2.15) than in children with TD (*M* = 16.25%, SD = 2.75), indicating higher methylation in the clinical group. No difference emerged for PC1 (*t* = 0.263, *p* = 0.794, Cohen’s *d* = 0.07) between children with NDs (*M* = 20.49%, SD = 2.48) and children with TD (*M* = 20.24%, SD = 4.05). As regards infant temperament, no significant differences emerged between the two groups. Descriptive statistics for the three domain of the IBQ-R/ECBQ are reported in Table 4.

**Table 4:** Descriptive statistics and *t*-test results comparing children with neurodevelopmental disabilities (NDs) and typically developing children (TD) across the three temperament traits assessed using the Infant Behavior Questionnaire-Revised (IBQ-R) (for children aged < 15 months) and Early Childhood Behavior Questionnaire (ECBQ) (for children aged ≥ 15 months).

Temperament traits	NDs		TD		*t*-test	
IBQ-R/ECBQ scores	*M*	DS	*M*	DS	*t* (46)	*p*
Surgency/Extraversion	4.21	1.26	4.31	0.89	−0.317	0.752
Negative emotionality	3.01	1.11	3.63	1.28	−1.79	0.079
Effortful control	4.95	0.73	5.18	0.67	−1.14	0.260

### Moderation models

Three moderation analyses were conducted to examine whether group moderates the relationship between *OXTR* DNAm PC2 and infant temperament traits, while controlling for child’s sex, age and DQ.

#### Surgency/Extraversion

**Figure 2:**
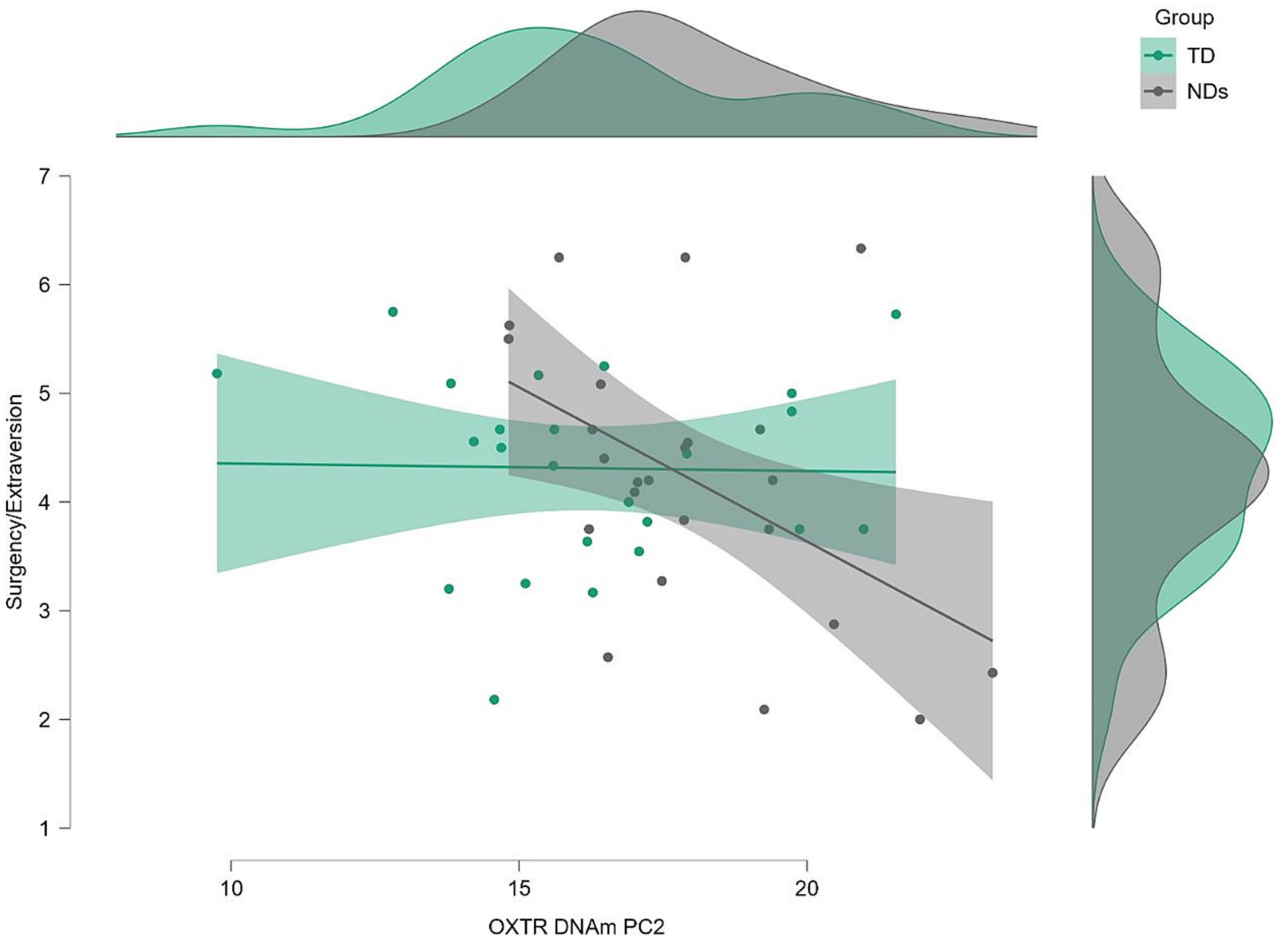
Moderation effect of group (NDs = children with neurodevelopmental disabilities; TD = typically developing children) between OXTR DNA methylation PC2 and child surgency/extraversion.

The overall model was statistically significant [*F* (6, 41) = 4.53, *p* = 0.001], accounting for 39.9% of the variance (*R^2^* = 0.399). The interaction between *OXTR* DNAm PC2 and group was significant [*b* = −0.30, SE = 0.11, *t* = −2.67, *p* = 0.011, 95% CI (−0.52, −0.07)], indicating that the effect of *OXTR* DNAm PC2 on Surgency/Extraversion differed depending on group. Simple slope analysis showed that for children with TD, the effect of *OXTR* DNAm PC2 on Surgency/Extraversion was non-significant [*b* = 0.02, SE = 0.07, *t* = 0.33, *p* = 0.746, 95% CI (−0.12, 0.16)]; for children with NDs, the effect was significant and negative [*b* = −0.27, SE = 0.09, t = −3.05, *p* = 0.004, 95% CI (−0.45, −0.09)]. This indicated that only for children with NDs, higher levels of *OXTR* DNAm PC2 were associated with lower Surgency/Extraversion. In contrast, for children with TD, *OXTR* DNAm PC2 did not significantly predict Surgency/Extraversion (Figure 1). Among the covariates, child age was a significant predictor of Surgency/Extraversion [*b* = 0.05, SE = 0.01, *t* = 3.77, *p* < 0.001, 95% CI (0.02, 0.08)], whereas sex [*b* = −0.32, SE = 0.27, *t* = −1.18, *p* = 0.24, 95% CI (−0.87, 0.23)] and DQ [*b* = 0.01, SE = 0.01, *t* = 0.85, *p* = 0.40, 95% CI (−0.01, 0.02)] were not (Figure 2).

#### Negative emotionality

The overall model was statistically significant [*F* (6, 41) = 4.53, *p* < 0.001], accounting for 56% of the variance (*R^2^* = 0.56); however, the interaction between *OXTR* DNAm PC2 and group was not significant [*b* = −0.15, SE = 0.11, *t* = −1.38, *p* = 0.175, 95% CI (−0.36, 0.07)], suggesting no group effect on the relation between *OXTR* DNAm PC2 and Negative Emotionality. Also, the moderation model did not yield main effects of both group and *OXTR* DNAm PC2. Among the covariates, child age [*b* = −0.07, SE = 0.01, *t* = −5.93, *p* < 0.001, 95% CI (−0.10, −0.05)] and DQ [*b* = 0.02, SE = 0.01, *t* = 2.60, *p* = 0.01, 95% CI (0.003, 0.03)] were significant predictors of Negative Emotionality, whereas sex was not [*b* = 0.15, SE = 0.26, *t* = 0.56, *p* = 0.58, 95% CI (−0.38, 0.68)].

#### Effortful control

The overall model was not statistically significant [*R^2^* = 0.21, *F* (6, 41) = 1.85, *p* = 0.116], indicating that neither *OXTR* DNAm PC2 and group nor their interaction predicted this temperament trait.

## Discussion

The present study aimed to investigate *OXTR* DNAm status in children with NDs and with TD and its potential associations with temperament traits. In line with the initial hypothesis, findings showed that children with NDs exhibited higher *OXTR* DNAm levels compared to TD children, suggesting a pattern of higher OXTR DNAmin this clinical group. A more detailed analysis identified significant differences in *OXTR* DNAm levels between the two groups, specifically in PC2 of the *OXTR* CpG sites. This component comprised several CpG sites within Intron 1, including CpG8 and CpG9, respectively at position −901 and −860 relative to the translation start site. Previous research has shown that increased DNAm at these two sites differentiates individuals with ASD from healthy controls across several tissues (Gregory et al., 2009). Although PCA-derived components are data-driven and sample-specific, PC2 may be interpreted as reflecting a pattern of co-varying CpG sites within this sample rather than a discrete functional regulatory unit. Our finding shows that DNAm is altered even in children with NDs, and this may suggest that variation in this CpG pattern (i.e., PC2, including CpG8 and CpG9) could be associated with impaired developmental profiles. This result is also consistent with the negative association observed between children’s developmental quotient scores and *OXTR* DNAm, suggesting that higher *OXTR* DNAm may be related to lower developmental functioning in children with NDs. At the same time, given that DQ is inherently linked to neurodevelopmental status, its inclusion in moderation models alongside group may entail partial over-adjustment. This conceptual overlap should be considered when interpreting these associations. Given that epigenetic variations of OXTR gene methylation have been related to reduced expression of the oxytocin receptor (Fineberg and Ross, 2017), these findings may be tentatively interpreted within the broader literature relating *OXTR* DNAm to o variation in oxytocin-related processes and socio-emotional and cognitive outcomes (Maud et al., 2018). In sum, our findings seem to suggest that a phenotype characterized by diminished developmental skills (i.e., NDs group) may be related, at least partially, to epigenetic modifications in the oxytocin system, although the specific biological interpretation of PC2 should be considered with caution. Additionally, we also found that higher levels of *OXTR* DNAm in PC2 were associated with lower Surgency/Extraversion, but only in children with NDs. Surgency/Extraversion is a temperamental trait marked by high activity levels, a strong desire for intense pleasure, low levels of shyness, and impulsivity (Rothbart and Putnam, 2002). Previous research showed that low levels of this trait are associated with reduced reciprocity during social interactions and lower social competence (Rubin et al., 2009; Dollar and Stifter, 2012). Children with lower level of Surgency/Extraversion are described as less engaged in social interaction, showing more passive behaviors, fewer spontaneous smiles, vocalizations, or physical gestures of enthusiasm (Dollar and Stifter, 2012). While their lower impulsivity may make them easier to manage in terms of self-regulation, their under-reactive social responsiveness may sometimes make it difficult for caregivers to interpret their social cues (Provenzi et al., 2020a, 2020b). In this sense, Surgency/Extraversion can be conceptualized as an early temperamental dimension that is expressed behaviorally in socio-emotional functioning, particularly in terms of social engagement and positive affect (Gartstein and Gonzalez, 2025). Although in the current study no difference emerged between children with NDs and with TD in Surgency/Extraversion, the association between *OXTR* DNAm and temperamental positive emotionality in children with NDs may reflect variability in these early behavioral expressions, rather than broader socio-emotional functioning per se (Giusti et al., 2018). Despite the absence of direct measures of socio-emotional behavior in the present study, this interpretation is consistent with prior literature linking Surgency/Extraversion to observable social engagement (Bornstein et al., 2015; Giesbrecht et al., 2022). Future research investigating the association between *OXTR* DNAm and infant temperament in clinical groups, and including direct assessments of socio-emotional functioning, is needed to further corroborate the current findings.

As for Negative Emotionality, although the model was statistically significant, neither main nor moderation effects were found for *OXTR* DNAm and group. Negative Emotionality is characterized by sadness, discomfort, frustration, fear, and difficulty to soothe (Kopala-Sibley et al., 2017). Children with higher level of negative emotionality tend to experience more intense and prolonged emotional reactivity to stressors and have difficulty recovering from peaks of distress (Laible et al., 2010). Overall, these findings indicate that *OXTR* DNAm was not significantly associated with Negative Emotionality in the present sample, regardless of developmental condition (NDs vs. TD). This result contrasts with previous research showing an association between OXTR hypermethylation and increased Negative Emotionality (Nazzari et al., 2024; Krol et al., 2019). This pattern may be interpreted as a developmental-stage hypothesis, whereby associations between *OXTR* DNAm and Negative Emotionality may differ across developmental stages, as prior studies focused on younger infants (e.g., 3- and 18-month-olds), whereas our sample included a broader age range (3–36 months). Accordingly, the association between *OXTR* DNAm and Negative Emotionality may be more evident earlier in development and less apparent at later stages. Consistent with this interpretation, age and DQ were negatively associated with Negative Emotionality in the present sample.

Finally, contrary to our expectation, the model for Effortful Control was not statistically significant as neither main nor moderation effects were found for *OXTR* DNAm and group. This result is consistent with a recent study that explored the epigenetic architecture of temperament in early infancy using a genome-wide approach (Leri et al., 2024), which found that Effortful Control does not yet exhibit a distinct epigenetic signature. In line with these findings, no association between OXTR DNAm and Effortful Control emerged in the present sample. One possible developmental interpretation is that Effortful Control, which involves higher-order cognitive processes such as attention regulation and inhibitory control, may show different patterns of association with DNAm across development. Accordingly, such associations may become more evident at later developmental stages, as neural systems supporting regulatory processes continue to mature (Wass, 2021). The present study has limitations to be acknowledged. First, the sample size is limited and future studies are needed to further confirm the relationship between *OXTR* DNAm and temperament in children with NDs. Studies with larger sample sizes may allow for stratification based on specific diagnoses with more homogeneous clinical characteristics (e.g., cerebral palsy, developmental delay), and the exploration of associations with specific phenotypic features. The limited sample size also has implications for the statistical analyses. Although an *a priori* power analysis was not conducted, the two significant moderation models explained a substantial proportion of variance in the outcome variables. These findings support the robustness and relevance of the observed effects, despite the constraints associated with the sample size. It is also important to note that the phenotypic variability within the clinical sample aligns with the typical heterogeneity observed in pediatric neurorehabilitation settings, where children often present with a broad spectrum of neurodevelopmental challenges. While this variability limits the possibility of examining more homogeneous diagnostic subgroups, it also enhances the ecological validity of the findings. Additionally, children with NDs were recruited in a clinical neurorehabilitation setting, whereas TD children were recruited from the community through convenience sampling. Although this recruitment strategy reflects common procedures in pediatric neurodevelopmental research, differences in recruitment context may introduce unmeasured environmental or psychosocial factors. Second, although it is well-established that children with NDs exhibit documented socio-emotional difficulties (Lipkin et al., 2020; Montirosso et al., 2025), we did not include a direct measurement of the child’s socio-emotional competencies in the present study. While temperament traits and oxytocin signaling have been related to social behavior (Poore and Waldman, 2020; Abulizi et al., 2017), any interpretation of the present findings in terms of broader socio-emotional outcomes remains speculative. Therefore, our results should be interpreted with caution and within the specific context of the temperament dimensions assessed. Future studies should consider incorporating direct assessments of socio-emotional skills to gain a more comprehensive understanding of the relationship between *OXTR* DNAm and socio-emotional development in children with NDs. Third, infant temperament was evaluated through a parent-report questionnaire completed by mothers. Although previous research has shown a reasonable alignment between parent reports and direct observations of infant temperament (e.g., Gartstein and Marmion, 2008; Stifter et al., 2008), future studies could enhance the assessment by including direct observational methods for a more thorough temperament evaluation. A related limitation concerns the use of two different instruments (IBQ-R and ECBQ) to assess temperament across age groups. Although both are grounded in the same theoretical model and yield comparable scores for the three main dimensions, structural differences between the questionnaires may introduce a heterogeneity. Fourth, the sample included children across a relatively broad developmental age range (3–36 months). This period encompasses rapid developmental changes in both socio-emotional functioning and the behavioral expression of temperament. Although age was examined in preliminary analyses and included as a covariate in the moderation models, developmental variability across this time window may still have influenced the observed associations. Fifth, DNAm levels were measured in peripheral tissue, specifically infant buccal epithelial cells collected through salivary swabs, and therefore represent an indirect proxy of OXTR expression in the brain. Nevertheless, previous research has documented substantial correspondence between genome-wide DNAm patterns observed in peripheral tissues and those found in human brain tissue. In particular, buccal epithelial cells appear to show DNAm profiles that are among the most comparable to those observed in the brain (Braun et al., 2019). Moreover, evidence suggests that OXTR DNAm profiles measured in saliva resemble those observed in brain tissue (Smith et al., 2015), and that saliva provides a reliable source for assessing OXTR methylation in humans (Krol et al., 2019). Taken together, these findings support the use of saliva-derived buccal cells as a feasible and non-invasive tissue for assessing OXTR methylation in early life. Sixth, while our research primarily examined epigenetic modifications of *OXTR*, previous studies have also linked DNAm of other genes to infant temperament (Leri et al., 2024). To gain a deeper understanding of how epigenetic modifications interact with genetic variations in shaping temperament differences, future studies should analyze additional genomic regions, and consider oxytocin gene polymorphisms. A further limitation of the present study is the lack of systematic data on other potential confounding factors known to influence both DNAm and child temperament. These include perinatal complications, medication use, maternal mental health, caregiving environment, socioeconomic conditions, and psychosocial adversity (Galbally et al., 2018; Maud et al., 2018; Unternaehrer et al., 2015, 2016). Although we controlled for DQ, which may partially capture broader developmental risk, future studies should incorporate more comprehensive assessments of biological, medical, and psychosocial factors. Including such data would allow for a more fine-grained analysis of how environmental exposures interact with epigenetic and neurodevelopmental mechanisms in shaping individual differences in temperament.

## Conclusion

Given the prevalence and significant impact of NDs, identifying potential early markers is crucial for enhancing diagnosis and enabling timely interventions. The current study suggests that epigenetic pathways, particularly *OXTR* DNAm, may contribute to variability in temperamental positive emotionality linked to NDs. Given that lower levels of Surgency/Extraversion are associated with difficulties in social interactions, these findings provide preliminary and exploratory evidence that *OXTR* DNAm epresent a potential epigenetic correlate of early socio-emotional development in this population. Future research is needed to explore this potential further and refine its application in early clinical settings. Moreover, the existing association between *OXTR* DNAm and Surgency/Extraversion provides initial evidence that *OXTR* DNAm may be associated with developmental variability in children with NDs.

## Data Availability

The datasets presented in this study can be found in online repositories. The names of the repository/repositories and accession number(s) can be found at: https://osf.io/pr5hk/overview, 10.17605/OSF.IO/PR5HK.

## Supplementary material

Supplementary Table S1 Demographic and clinical features of the subgroups with developmental delay and with cerebral palsy.

Supplementary Figure S1 Developmental Quotient (DQ) in children with neurodevelopmental disabilities (ND) and typically developing (TD) children. Preliminary analyses

Supplementary Table S2 OXTR DNAm levels across CpG sites and subgroup comparisons (Cerebral Palsy vs. Developmental Delay).

Supplementary Table S3 False Discovery Rate (FDR) correction using the Benjamini–Hochberg procedure.
