# Supplementary material for: Exploring the relationship between OXTR DNA methylation and temperament in children with neurodevelopmental disabilities
Source: Front Psychol. 2026 Apr 15;17:1763938. doi: 10.3389/fpsyg.2026.1763938 (PMC13124945; doi:10.3389/fpsyg.2026.1763938)
Supplement: Supplementary Table S1 — Demographic and clinical features of the subgroups with developmental delay and with cerebral palsy. [file Table_1.docx]

Mascheroni et al

**Exploring the Relationship Between *OXTR* DNA Methylation and Temperament**

**in Children with Neurodevelopmental Disabilities**

**SUPPLEMENTARY MATERIALS**

**Supplementary Table S1** | page 2

**Supplementary Figure S1** | page 3

**Supplementary Preliminary analyses** | page 4

**Supplementary Table S2** | page 4

**Supplementary Table S3** | page 5

**Supplementary Table S1**. Demographic and clinical features of the subgroups with developmental delay and with cerebral palsy.

|  | **Developmental delay** | **Cerebral palsy** |
| --- | --- | --- |
| **Sample caractheristics** - Mean (SD)/N (%) |  |  |
| Sex (females) | 7 (58%) | 5 (42%) |
| Child age (months) | 20.1 (7.1) | 17.8 (10.3) |
| Maternal age (years) | 37.3 (5.1) | 33.8 (5.8) |
| Child Developmental Quotient (score) | 55.1 (24.7) | 75.3 (24.7) |
| **Clinical signs** (N) |  |  |
| MRI | Cortical and subcortical abnormalities (5); none (5); not available (2) | Periventricular leukomalacia (3); focal white matter damage (3); multiple cortical and subcortical injuries (4); post-hemorrhagic hydrocephalus (1); focal white and gray matter damage (1) |
| EEG | Multifocal epileptic abnormalities (3); sporadic epileptic abnormalities (1); irregular activity but no epileptic signs (3); not available (5) | Multifocal epileptic abnormalities (2); sporadic focal epileptic abnormalities (1); asymmetric/irregular activity but no epileptic signs (3); not available (6) |
| **Diagnosis** | Congenital encephalopaty (2); methylmalonic aciduria (1); DD in congenital brain malformations (3); DD in genetic defects (1); DD with prevalent language impariments (2); Unknown (3) | Bilateral CP (6); unilateral CP (6) |

**Supplementary Figure S1**. Developmental Quotient (DQ) in children with neurodevelopmental disabilities (ND) and typically developing (TD) children*. Boxplots show the distribution of Griffiths III – Total Developmental Quotient (DQ) scores in the two groups. The central line indicates the median, the box represents the interquartile range (IQR), and the whiskers extend to 1.5 × IQR. Individual data points are overlaid to display the full distribution of scores in each group. The horizontal blue line at 100 represents the normative mean, while the dashed lines at 85 and 115 indicate ±1 standard deviation from the mean. TD children show higher DQ scores compared to children with neurodevelopmental disorders (t (46) = -7.18, p < .001).*

**
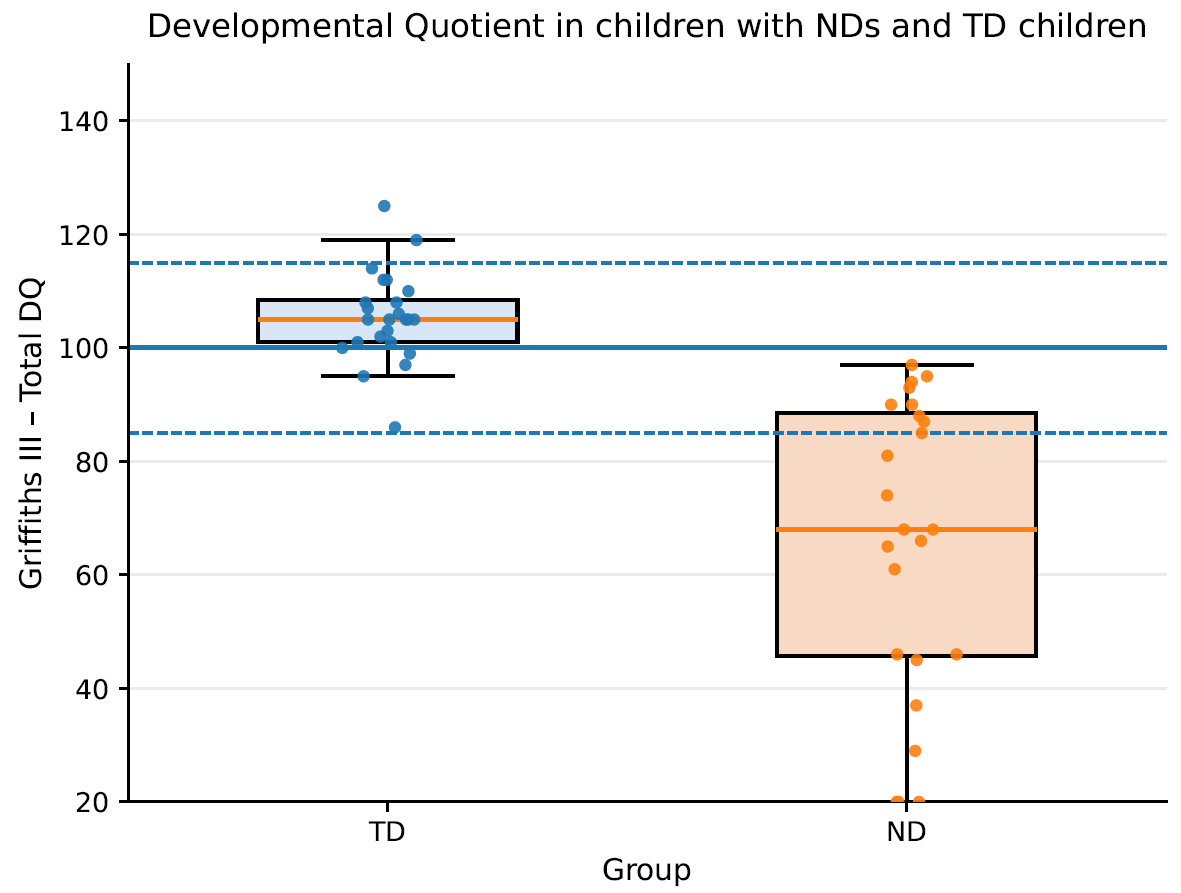
**

**Preliminary analyses**

No significant differences were found between the NDs and TD groups in terms of sex distribution (*χ²* = 0.336, *p* = .562) and age (*t* (46) = 0.879, *p* = .384). Differences were found in DQ scores (*t* (46) = -7.18, *p* < .001). In a similar vein, the two clinical subgroups (Cerebral Palsy vs Developmental Delay) did not differ in terms of biological sex distribution (*χ²* = 0.67, *p* = .414) and age (*t* (22) = -0.625, *p* = .539). Mann–Whitney U tests revealed no significant differences between the two clinical subgroups in *OXTR* DNAm principal components (PC1: U = 64.00, *p* = .644; PC2: U = 69.00, *p* = .862). Descriptive statistics and subgroup comparisons for DNAm levels across the 13 CpG sites are reported in Supplementary Table S2.

**Supplementary Table S2**. *OXTR* DNAm levels across CpG sites and subgroup comparisons (Cerebral Palsy vs Developmental Delay)

| **CpG site** | **Position** | **Mean ± SD** | **Min–Max** | **U** | **p** |
| --- | --- | --- | --- | --- | --- |
| CpG1 | chr3: 8810889–8810890 | 1.37 ± 0.91 | 0.08–4.13 | 71.00 | .954 |
| CpG2 | chr3: 8810874–8810875 | 2.16 ± 1.02 | 0.08–4.38 | 43.00 | .094 |
| CpG3 | chr3: 8810862–8810863 | 6.34 ± 2.39 | 0.57–11.46 | 54.50 | .312 |
| CpG4 | chr3: 8810855–8810856 | 1.79 ± 1.04 | 0.08–4.21 | 71.00 | .954 |
| CpG5 | chr3: 8810832–8810833 | 31.17 ± 4.45 | 24.28–43.28 | 59.00 | .453 |
| CpG6 | chr3: 8810807–8810808 | 35.39 ± 5.71 | 22.25–44.39 | 59.00 | .453 |
| CpG7 | chr3: 8810797–8810798 | 60.66 ± 6.04 | 44.29–70.49 | 64.00 | .644 |
| CpG8 | chr3: 8810774–8810775 | 43.81 ± 4.64 | 33.25–49.97 | 71.00 | .954 |
| CpG9 | chr3: 8810733–8810734 | 24.22 ± 3.89 | 18.42–32.96 | 54.00 | .299 |
| CpG10 | chr3: 8810708–8810709 | 8.86 ± 2.65 | 4.17–15.42 | 62.00 | .564 |
| CpG11 | chr3: 8810699–8810700 | 9.89 ± 3.41 | 4.22–16.46 | 69.00 | .862 |
| CpG12 | chr3: 8810681–8810682 | 11.08 ± 3.43 | 3.19–15.88 | 65.50 | .707 |
| CpG13 | chr3: 8810679–8810680 | 12.04 ± 3.42 | 6.43–20.04 | 49.00 | .184 |

Similarly, no subgroup differences emerged in temperament traits (Negative Emotionality: U = 58.00, *p* = .419; Surgency/Extraversion: U = 66.50, *p* = .751; Effortful Control: U = 63.50, *p* = .623). Preliminary *t*-tests also revealed no differences between males and females in terms of *OXTR* DNAm (PC1: *t* (46) = 1.81, *p* = .077; PC2: *t* (46) = 1.63, *p* = .111) and temperament (Surgency/Extraversion: *t* (46) = -1.45, *p* = .130; Negative Emotionality: *t* (46) = .489, *p* = .627, Effortful control: *t* (46) = -1.86, *p* = .070). Moreover, preliminary Pearson’s correlations showed no significant relationship between age and *OXTR* DNAm (PC1: *r* = 0.060, *p* = .685; PC2: *r* = 0.052, *p* = .726); significant associations between age and child temperament emerged for Surgency/Extraversion (*r* = .424, *p* = .003) and Negative Emotionality (*r* = -.656, *p* < .001), but not for Effortful Control (*r* = -.267, *p* = .066). The DQ was significantly negatively correlated with *OXTR* DNAm PC2 (*r* = -0.325, *p* = .024) but not with *OXTR* DNAm PC1 (*r* = -0.16, *p* = 0.227); DQ also significantly correlated with Negative Emotionality (*r* = .374, *p* = .009), but not with Surgency/Extraversion (*r* = .136, *p* = .357) and Effortful Control (*r* = .057, *p* = .700).

**Supplementary Table S3.** False Discovery Rate (FDR) correction using the Benjamini–Hochberg procedure. *Uncorrected and FDR-adjusted p-values are reported for the main hypothesis-driven analyses, including group comparisons (ND vs TD) on principal components (PC1 and PC2), moderation models testing the interaction between OXTR DNAm PC2 and group on temperament dimensions, simple slope analyses, and the association between developmental quotient (DQ) and PC2.*

| **Analysis** | ***p* (uncorrected)** | ***p* FDR** |
| --- | --- | --- |
| ND vs TD difference – PC2 | 0.019 | 0.038 |
| ND vs TD difference – PC1 | 0.794 | 0.794 |
| PC2 × group interaction (Surgency) | 0.011 | 0.029 |
| Simple slope PC2 → Surgency (ND) | 0.004 | 0.016 |
| Simple slope PC2 → Surgency (TD) | 0.746 | 0.794 |
| PC2 × group interaction (Negative Emotionality) | 0.175 | 0.233 |
| Overall model – Negative Emotionality | 0.001 | 0.008 |
| Correlation DQ – PC2 | 0.024 | 0.038 |
